# Developing Aboriginal and Torres Strait Islander cultural indicators: an overview from Mayi Kuwayu, the National Study of Aboriginal and Torres Strait Islander Wellbeing

**DOI:** 10.1186/s12939-022-01710-8

**Published:** 2022-08-17

**Authors:** Sarah C. Bourke, Janet Chapman, Roxanne Jones, Makayla-May Brinckley, Katherine A. Thurber, Bianca Calabria, Kate Doery, Anna Olsen, Raymond Lovett

**Affiliations:** 1 National Centre for Epidemiology and Population Health, College of Health & Medicine, Australian National University, Canberra, Australia; 2 School of Anthropology and Museum Ethnography, University of Oxford, Oxford, UK; 3 College of Health & Medicine, Australian National University, Canberra, Australia; 4 National Drug and Alcohol Research Centre, University of New South Wales, Sydney, Australia; 5 The Teal Psychology Space, Canberra, Australia; 6 Centre for Social Research and Methods, Research School of Social Sciences, Australian National University, Canberra, Australia; 7 Policy and Equity, Centre for Community Child Health, Murdoch Children’s Research Institute, Royal Children’s Hospital, Melbourne, Australia; 8 Medical School, College of Health & Medicine, Australian National University, Canberra, Australia

**Keywords:** Culture, Aboriginal and Torres Strait Islander, Domain development, Questionnaire item development, Survey research

## Abstract

**Background:**

For Aboriginal and Torres Strait Islander peoples, culture is foundational to health and wellbeing. However, its inherent conceptual complexity and diversity across and within different Aboriginal and Torres Strait Islander cultural groups means that it has rarely been explored in depth by epidemiological research. As a result, there are very few measures which adequately represent the heterogeneity and importance of Aboriginal and Torres Strait Islander cultures for health and wellbeing. Tools grounded in the social determinants of health are mostly based on European academic opinion about what constitutes culture and wellbeing, and the views of Indigenous peoples are rarely included. Mayi Kuwayu, the National Study of Aboriginal and Torres Strait Islander Wellbeing, developed a new survey tool based on health and wellbeing as perceived by Aboriginal and Torres Strait Islander people. This paper describes several of the key processes used to identify cultural domains and develop questionnaire items for the survey tool, reflecting the importance of culture to Aboriginal and Torres Strait Islander peoples.

**Methods:**

Focus groups were conducted at community organisations and conferences with Aboriginal and Torres Strait Islander people. These sessions were aimed at identifying key cultural domains to be addressed by the Mayi Kuwayu questionnaire and to field test drafts of the questionnaire, which were then modified according to focus group feedback and expert input.

**Results:**

Extensive community consultations allowed us to identify key cultural domains, generate questionnaire items, and test initial content validity. The six overarching cultural domains identified during the development of the Mayi Kuwayu questionnaire were: Connection to Country; Beliefs and knowledge; Language; Family, kinship, and community; Cultural expression and continuity; and Self-determination and leadership.

**Conclusions:**

The processes used by Mayi Kuwayu have generated meaningful cultural items for use in Aboriginal and Torres Strait Islander health and wellbeing research. Further assessment of these processes, including a comparison with best practice guidelines and psychometric testing of the items and scales developed, will be conducted in a future program of work.

**Supplementary Information:**

The online version contains supplementary material available at 10.1186/s12939-022-01710-8.

## Introduction

Aboriginal and Torres Strait Islander peoples represent two distinct populations with great diversity in cultures and practices both between and within these groupings [1, 2] which have been continued, maintained, and modified in Australia for at least 65,000 years [3, 4]. Colonial processes and government policies and practices have deliberately and negatively impacted Aboriginal and Torres Strait Islander peoples [5–7]. Aboriginal and Torres Strait Islander communities have worked to preserve traditional forms of culture, alongside creating new forms of expression, demonstrating strength and resilience in the face of ongoing colonial practices and mindsets in Australia [5]. The potential benefits of being able to sustain a strong cultural identity has been documented in settings across the world [1, 8–11]. There is evidence from Australia and Canada that engagement in cultural revitalisation or renewal activities fosters cultural belonging and can mediate or reverse the effects of intergenerational trauma [12–16]. Reflecting on this work, Aboriginal and Torres Strait Islander cultural identity is central to the Social and Emotional Wellbeing framework of health in Australia [17]. Yet, the theory that Aboriginal and Torres Strait Islander cultural identity is linked to health and wellbeing has not been tested empirically.

There is a lack of research understanding on what culture means from an Aboriginal and Torres Strait Islander standpoint and how this concept is tied to health outcomes. Drawing on the epistemological approaches of epidemiology paired with a de-colonising lens, a national study was developed to define Aboriginal and Torres Strait Islander culture and wellbeing from this *emic* perspective. Mayi Kuwayu, the National Study of Aboriginal and Torres Strait Islander Wellbeing, is now Australia’s largest longitudinal study of Aboriginal and Torres Strait Islander wellbeing.

This paper focuses on the initial work undertaken by the Mayi Kuwayu team to define concepts of culture and measures of wellbeing. It provides an overview of the community focus groups conducted by and with Aboriginal and Torres Strait Islander people to identify key cultural domains which influence health and wellbeing outcomes and refine the study’s questionnaire. The Mayi Kuwayu questionnaire took four years to develop from conception through to release of the questionnaire in 2018. The pragmatic nature of the processes used draw attention to the ways in which common methods in public health can be adapted to Aboriginal and Torres Strait Islander settings, and how community validation of research methods is a key part of conducting research that is relevant and of benefit to Aboriginal and Torres Strait Islander peoples.

### Measuring culture

Identifying the broad concepts or domains of culture that influence health outcomes, and developing questionnaire items to capture experiences within these domains, are important for quantifying population trends, monitoring changes, and evaluating the impact of domains on quality of life and wellbeing [18, 19]. To fully understand wellbeing for Aboriginal and Torres Strait Islander people, cultural items are required to measure the breadth of shared cultural attributes and generate relevant large scale data [20].

Mayi Kuwayu was developed to address this need and is the first large-scale, longitudinal, comprehensive examination of the link between culture and wellbeing for Aboriginal and Torres Strait Islander peoples [21]. The main component of the study is a questionnaire which includes items on cultural practice and expression, sociodemographic factors, health and wellbeing, health behaviours, experiences and environments, and family support and connection [21]. This questionnaire was first mailed out in 2018 to over 200,000 Aboriginal and Torres Strait Islander people aged 16 years and older, as identified in Australia’s Medicare database, and is also available to complete online through the study’s website (www.mkstudy.com.au). There are now over 11,000 Mayi Kuwayu participants, making this the largest cohort study of Aboriginal and Torres Strait Islander health and wellbeing in Australia [22].

The study has been led, developed, conducted, and governed by Aboriginal and Torres Strait Islander people, and includes direct and ongoing involvement from external Aboriginal and Torres Strait Islander researchers, communities, and organisations across Australia [21]. Mayi Kuwayu has a governance group which includes several peak Aboriginal and Torres Strait Islander health and research groups, including the National Aboriginal Community Controlled Health Organisation, and State and Territory affiliate organisations. Its data governance processes include an all-Indigenous Data Governance Committee that applies the Maiam nayri Wingara Indigenous Data Sovereignty principles [23] to assess data use requests, along with continued engagement with communities in the implementation of the questionnaire, and the analysis, interpretation, and dissemination of data collected.

In 2017, an international literature review was undertaken by members of the Mayi Kuwayu team to identify key domains (and any additional sub-domains) of Indigenous cultures which had relationships to health and wellbeing outcomes [2]. The review identified six broad domains within culture, each with several sub-domains:

1. Connection to Country – including spiritual connection, health, and traditional foods, living on Country, land rights and autonomy, caring for Country, and impacts of tourism.
2. Beliefs and knowledge – including spiritual and religious beliefs, traditional knowledge, traditional healing, and knowledge transmission and continuity.
3. Language – including impacts of language on health, language revitalisation, and Indigenous language education.
4. Family, kinship, and community – including family and kinship, community, sport, and social determinants of health.
5. Cultural expression and continuity – including identity, cultural practices, arts, and music.
6. Self-determination and leadership – including cultural safety, self-determination and wellbeing, and leadership.

The aim of this paper is to highlight how the Mayi Kuwayu questionnaire was developed, contributing to the limited published literature in this space, and particularly, epidemiological work ontologically situated in an Indigenous and de-colonising framework. This paper provides an overview of the community-based focus groups and how they contributed towards refining questionnaire items alongside expert input to create ‘good data’ which advance Indigenous data sovereignty and governance objectives [24].

## Methods

### Focus groups

#### Recruitment and participation

The Mayi Kuwayu team approached Aboriginal and Torres Strait Islander community organisations through the study’s governance group and provided information about the study, its goals, and its desire to conduct community-based focus groups. Aboriginal and Torres Strait Islander community organisations then self-nominated to facilitate or co-facilitate a focus group. Community organisations included health services and partner organisations of the study (e.g. Aboriginal and Torres Strait Islander community-controlled health, sporting, and land rights organisations, Aboriginal and Torres Strait Islander Research Centres, running groups, and neighbourhood centres). Participating community organisations were located nationally, across saltwater/freshwater/desert/Island groups, and in urban, regional, and remote areas, representing the high degree of diversity in cultural experiences. Their locations included areas where Aboriginal and Torres Strait Islander people were the minority or majority of the population, and had experienced varied impacts of colonisation, including forced removals (e.g. missions and Stolen Generations). The community organisations were compensated with AUD$5,000 for the time and resources they used to organise the focus groups. The Mayi Kuwayu team also hosted focus groups at two national conferences where there were large numbers of Aboriginal and Torres Strait Islander attendees. The study conducted 28 focus groups in total, reflecting the importance of ensuring that our questionnaire was able to capture the diversity of Aboriginal and Torres Strait Islander cultures and be applicable and meaningful across multiple contexts. We used an iterative process to determine key cultural domains and refine the questionnaire across the focus groups (see Results).

#### Procedure for focus groups at community organisations

Focus groups at community organisations were led by an Aboriginal Mayi Kuwayu team member and co-facilitated by local staff or a community member when requested by the participants. Each focus group was between one and four hours in duration, depending on the level and type of engagement (e.g. some focus groups were longer when participants shared personal or cultural stories). Cultural protocols for focus group participation were considered and guided by the community organisation. For example, on occasion there needed to be gender-specific focus groups to adhere to cultural norms within these communities, including having facilitators whose gender matched with the participants who were present.

At the commencement of each focus group, facilitators acknowledged Country and Traditional Owners and then introduced themselves, including which cultural group/s they belonged to and their professional background. This practice was used to help participants feel more comfortable and confident talking with the Mayi Kuwayu team member who shared an Indigenous lived experience with them. The facilitators would then describe the purpose and aims of the focus group and explain the informed consent process. This included the confidentiality of the focus group and that the reporting of any work from the group would be de-identified to help facilitate open discussion.

After the consent forms were completed, the participants were asked if an audio recorder may be used for the session. If any participant did not consent to audio recording, the session was not recorded, and the facilitators took notes of the general themes. In addition to the focus groups being recorded (where consent was provided), other aids were sometimes used, such as butchers’ paper and whiteboards to write down key terms. Participants were also asked not to discuss any issues which may be sensitive and to identify any topics which should not be included in the research (e.g. women’s or men’s business). Anything deemed culturally inappropriate by the participants was not included in the researcher’s notes or audio recordings. If anything culturally inappropriate was breached during the discussion, then participants could approach the facilitator and have a conversation away from the group where it would not be recorded. All audio recordings were then professionally transcribed before being manually reviewed by the researchers to identify key cultural domains.

The focus groups were conducted as a free-flowing conversation with participants interacting with one another, and the facilitators occasionally guiding the group when necessary. All participants were encouraged to contribute to the discussion but were told that if they did not feel comfortable answering specific questions they did not have to contribute. Each focus group followed a similar structure and was divided into two parts: 1) discussing culture and its meaning for the participants, and 2) pre-testing the questionnaire.

The aim of the first part of the focus group was to understand what Aboriginal and/or Torres Strait Islander culture was for the participants. In the initial focus group sessions conducted in regional New South Wales (Groups 1–5) the facilitators asked numerous questions about different aspects of culture. First, they asked “Can you tell me about the important things that you think make up Aboriginal and Torres Strait Islander culture?”. When a participant provided a response, they were then asked follow-up questions such as: “Can you tell me a bit more about that (domain)? How do you learn about this (domain)? Where does it come from? Why is this important? Can you give me an example of why this (specific element: specific scenario or part of the broader domain) is important? How would you describe (specific element) to others?”. For subsequent sessions these were refined into two key questions used to lead the discussion:

1. Can you tell me about the important things that you think make up Aboriginal and Torres Strait Islander culture here?
2. How do you see culture influencing your health and wellbeing?

Before ending the discussion, a final question was asked to ensure any other important information was not excluded: “Is there anything else that anyone feels we should have talked about but didn’t?”.

The aim of the second part of the focus group was to pre-test the questionnaire and was not audio recorded. Questionnaires were handed out to participants who provided consent to have their answers used for Mayi Kuwayu research analyses and publications. They were also given the option for data linkage in future studies. Paper versions of the questionnaire were self-completed by participants with facilitators available to answer any queries. The facilitators were also available to support completion of the questionnaire in an interview format when required or requested by participants. Participants were encouraged to provide feedback on the questionnaire as a whole and on specific items and themes. With each new focus group session, the questionnaire was developed and refined by the Mayi Kuwayu team to incorporate feedback and to reflect the key cultural domains brought up during the discussions.

#### Procedure for focus groups at conferences

Focus group sessions were conducted at two national conferences and followed a similar format to the community focus groups. These focus groups were organised as a session held during the conference and advertised as a research session that Aboriginal and/or Torres Strait Islander attendees could participate in to develop a questionnaire of culture and health. For these sessions several tables were set up in a meeting room, with information and consent forms placed in front of each seat, and butchers’ paper and pens positioned at the centre of the table. A PowerPoint presentation was set up at the front of the room, which the Mayi Kuwayu team used to deliver their introductions and information about the study to the audience. Each table had between four and 10 participants depending on the space available, with each table representing a separate focus group.

At the commencement of these sessions, an Aboriginal Mayi Kuwayu team member or conference facilitator acknowledged Country and Traditional Owners and then introduced themselves, including which cultural group/s they belonged to and their professional background. Information about Mayi Kuwayu, including the protocols for confidentiality and consent were discussed, and attendees were asked to complete a consent form if they agreed to participate. Non-Indigenous conference attendees were permitted to observe the focus groups with the permission of the Aboriginal and Torres Strait Islander attendees but did not participate in the discussions. Only the responses from Aboriginal and Torres Strait Islander participants were included for analysis by the Mayi Kuwayu researchers.

The Mayi Kuwayu team member leading the sessions asked the groups to discuss one key question amongst themselves: “What are the things that make up Aboriginal/Torres Strait Islander culture?” with 20–30 min allocated for this discussion. A scribe was elected within the group to record a summary of the group’s responses on butchers’ paper. At the completion of the exercise a representative from each group presented their main points to the rest of the room. The sessions were not audio recorded for practical reasons. The groups responses were then collected by the Mayi Kuwayu team and manually analysed to inform the development of the cultural domains. Following this exercise, the participants were invited to complete a draft of the questionnaire and told to request help from the facilitators if they had any queries. The main queries from these focus groups concerned clarifying the meaning of some items.

## Results

### Questionnaire refinement

#### Participant summary

Twenty-eight focus groups were conducted by Mayi Kuwayu team members across Australia between 2015 and 2017, with a total of 197 participants. As shown in Table 1, the national conference focus groups accounted for 10 of the 28 focus groups (35.7%). In each of the 28 focus groups there were between three and 13 participants aged 16 years and over. The majority of the participants were middle-aged (35–55 years old) and lived in regional areas, and there was a general under-representation of younger people (aged 16–24 years old) and residents of urban areas compared to the national population distribution. A summary of the focus group locations and number of participants is shown in Table 1.

**Table 1:** Summary of Mayi Kuwayu focus groups

Focus Group	State/Territory	Location (Urban, Regional, Remote)	Type of Organisation	Number of Participants
1	NSW	Regional	Land Councils, NGO, ACCHO	25
2				
3				
4				
5	NSW	Regional	NGO	9
6	NT	Remote	Land Council	7
7	NSW	Regional	Land Council	9
8	ACT	N/A	National Conference	25
9				
10				
11	NSW	Regional	NGO	7
12	WA	Regional	ACCHO	8
13				13
14	QLD	Regional	ACCHO	4
15	QLD	Remote	Sporting Organisation	5
16	NT	Regional	NGO	4
17	SA	Remote	NGO	7
18	ACT	Urban	NGO	5
19	WA	Regional	ACCHO	10
20	WA	N/A	National Conference	6
21				9
22				5
23				7
24				8
25				4
26				9
27	TAS	Urban	NGO	8
28	NT	Remote	NGO	3
Total Number of Participants				197

*NSW* New South Wales, *NT* Northern Territory, *ACT* Australian Capital Territory, *WA* Western Australia, *QLD* Queensland, *SA* South Australia, *TAS* Tasmania, *NGO* Non-Government Organisation, *ACCHO* Aboriginal Community Controlled Health Organisation

#### Analysis of focus group discussions and questionnaire refinement

An iterative process was used by the Mayi Kuwayu team to refine the cultural domains and questionnaire item pool for each subsequent focus group session. The team met regularly to review focus group feedback and amend the questionnaire. The process overwhelmingly involved suggestions from the participants to amend the wording of items to improve comprehension by or relevance for the participants. Clarifying terms were particularly important for cultural terminology to optimise understanding across diverse Aboriginal and Torres Strait Islander groups. For example, feedback received from a focus group conducted in remote Queensland resulted in the questionnaire including “Where your Country/Island?” rather than “Where is your Country?”. Another example was the change from asking participants “What is your skin name?” and “What is your totem/dreaming?” to instead ask “Do you know your skin name(s)?” and “Do you know your totem(s)/dreaming?”. This was to ensure that cultural sensitivities were catered for, as some focus group participants explained that expressing skin and totemic names was sometimes not appropriate for secret/sacred reasons.

**Fig. 1:**
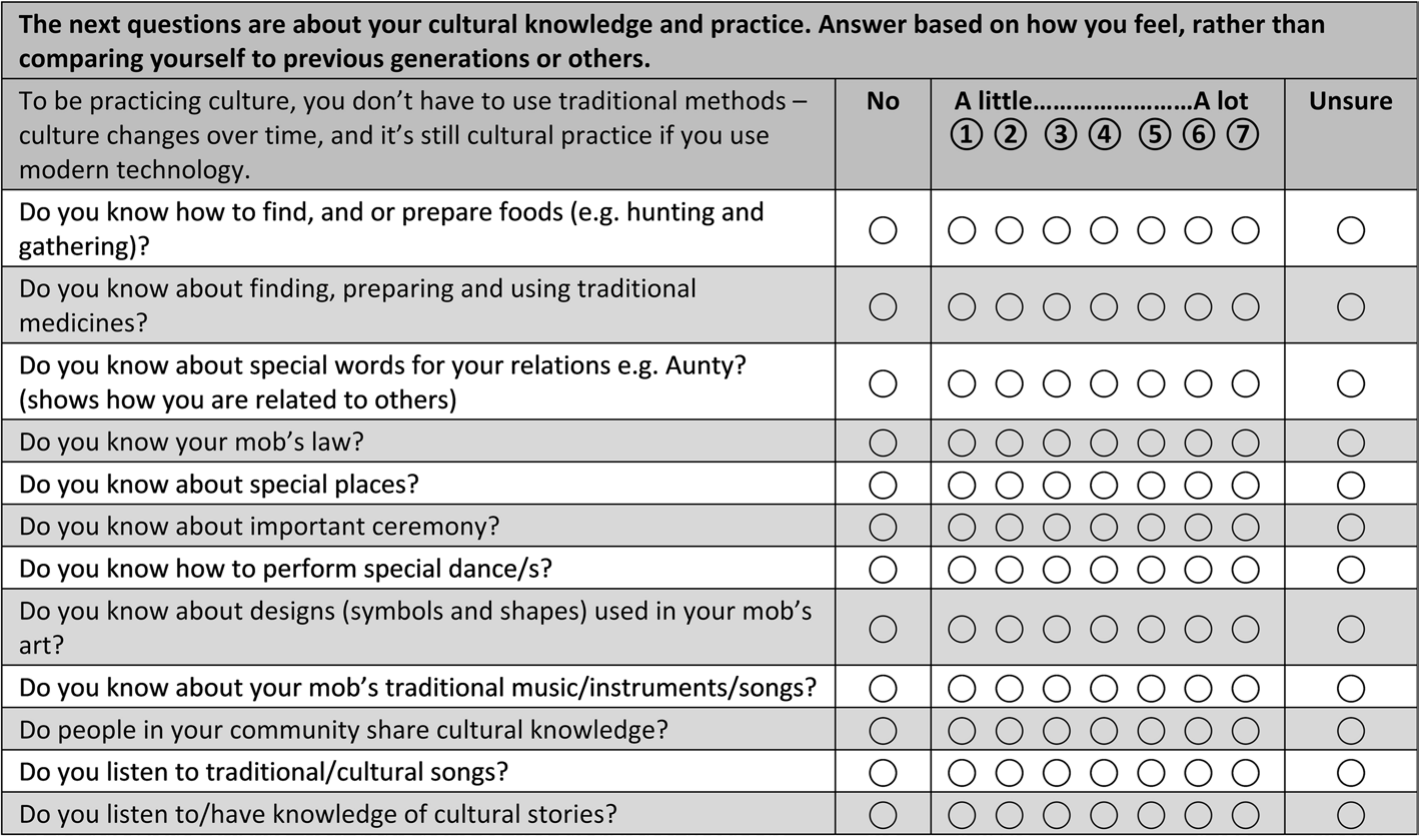
Cultural knowledge and practice section from initial focus groups

The *Cultural knowledge and practice* section of the questionnaire described below has been included in every draft used for the focus groups and incorporates a range of cultural items. The process used to refine it is representative of how other items were refined over time. Figure 1 shows how this section appeared in the initial focus groups. The phrasing of the first two lines in Fig. 1 addresses pervasive White Australian views of Indigenous authenticity, which can be internalised by people who feel like they are ‘not Aboriginal enough’ if they do not have traditional cultural knowledge or practices (for a critical discussion of this phenomenon, see [25]). The wording above counters this deficit discourse by emphasising a strengths-based message: ‘To be practicing culture, you don’t have to use traditional methods – culture changes over time, and it’s still cultural practice if you use modern technology’.

**Fig. 2:**
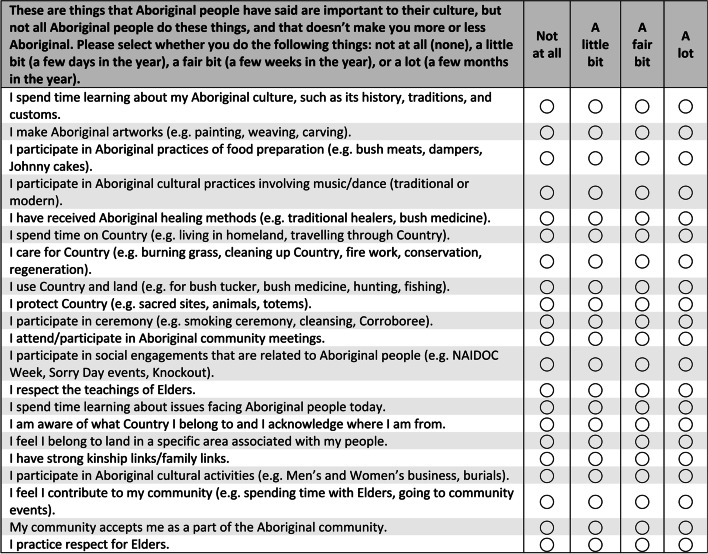
Cultural knowledge and practice section used in groups 12 and 13

**Fig. 3:**
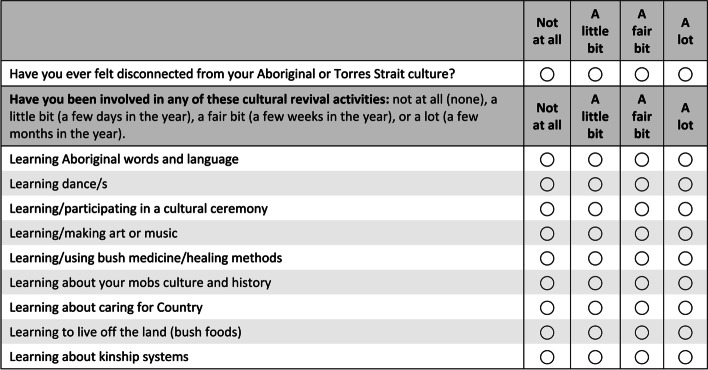
Additional questions added for groups 14 and 15

By focus groups 12 and 13 there were 21 cultural items in the *Cultural knowledge and practice* section (see Fig. 2), compared to the 12 items used previously. Most were added following focus group feedback to include the different ways people connect to Country. This exemplifies the inductive nature of our questionnaire development where some items became longer before they were later refined. Halfway through the focus group consultations, references to ‘Aboriginal’ were replaced with ‘Aboriginal/Torres Strait Islander’ as more Torres Strait Islander participants became involved. Changes were also made to the format of the *Cultural knowledge and practice* section to address experiences of cultural disconnection and cultural revival activities (Fig. 3).

**Fig. 4:**
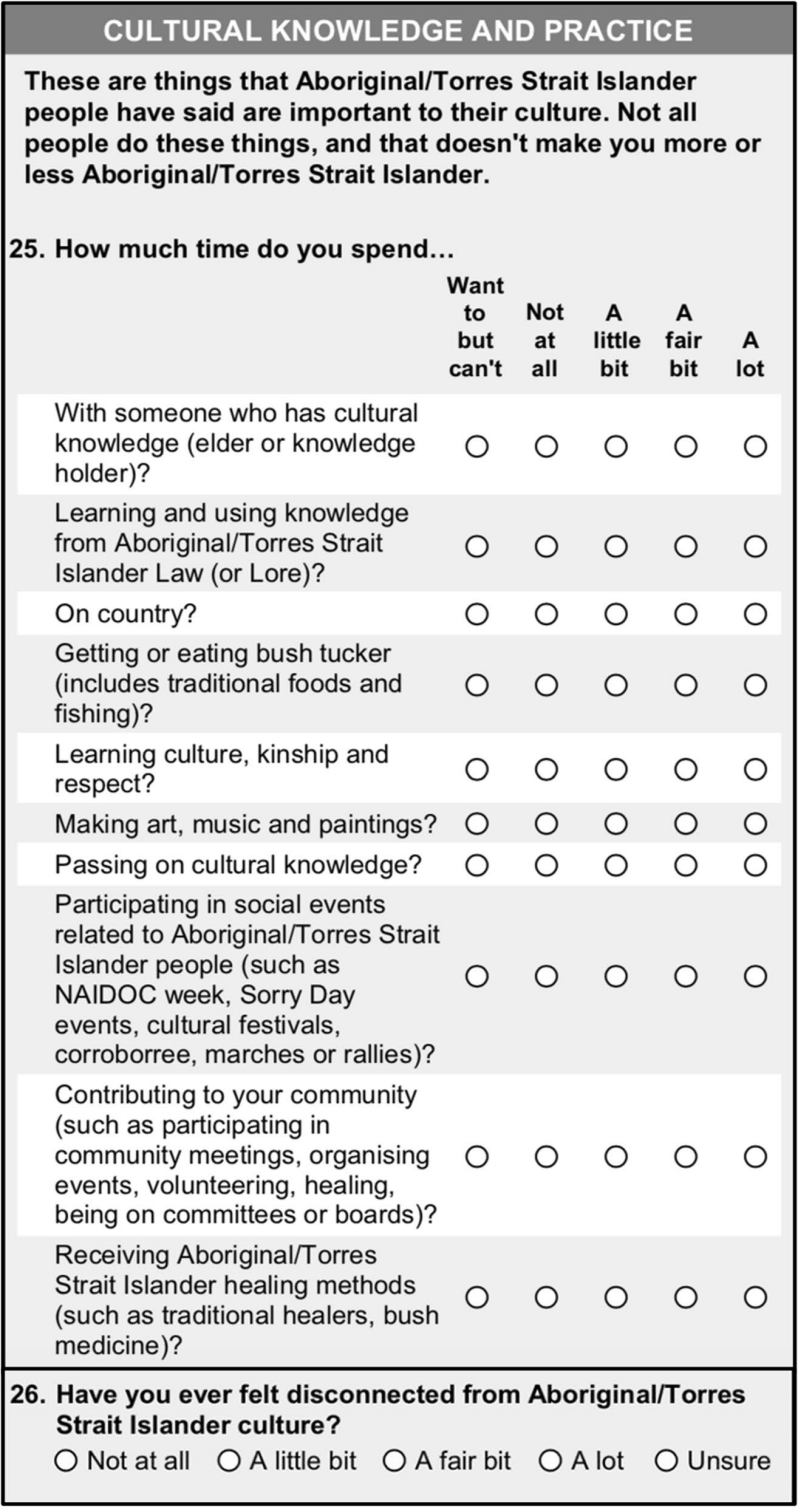
Cultural knowledge and practice section from the final questionnaire

After the focus group consultations had been conducted, expert evaluations were used to further refine the questionnaire content. The final version of the *Cultural knowledge and practice section* is shown in Fig. 4.

Table 2 provides other examples of how specific items in the questionnaire were developed for three of the six cultural domains discussed during the focus groups.

**Table 2:** Examples of how focus group feedback was incorporated into the Mayi Kuwayu questionnaire

Cultural Domain	Extracts from Focus Groups	Cultural Item/s
Connection to Country	“When we go up on Country, it’s about taking the kids through the landscape, talking to them about special significant sites and what happened and showing them the fish traps and ground ovens and all those sorts of things. So sharing that understanding is not just having a connection to your land but actually understanding their lands is really important.” “Part of our role is taking people out to Country, showing them Country, just to get away from the community life and away from town as well… It does get some positive energy and builds their strength when they go out on traditional land and stuff like that. And drinking water from their waterholes and bush tucker from out bush, and all that. That’s what builds people’s strength up.”	Q25. How much time do you spend… -On Country? -Getting or eating bush tucker (includes traditional foods and fishing)? -Passing on cultural knowledge?
Language	“Language is our knowledge. Our power to express ourselves.” “I just think language, when you speak it, it’s like… It’s like a song when you’re speaking it… that feeling you get when you hear people speaking it. I don’t know, can’t describe it but that’s one of the positives… that’s growing language and sharing.”	Q23. Tell us about your Aboriginal/Torres Strait Islander words or language -It is important that I use words or language -I feel good when I use words or language -I am learning words or language -My community is interested in keeping language strong
Cultural expression and continuity	“Culture for me is respecting our elders. By means of that is that they are our first teachers. They’re our guidance. They’re our backbone of our family… help us connect back into Country. They teach us knowledge, history, storylines, songlines.” “And it’s all about passing on that knowledge. Cultural knowledge… and taking care of passing on that information.” “We’re trying to get the younger ones to recognise which plants are medicine, which are the drinking ones, which are the rubbing ones, which ones are antiseptic. ‘Cause they’re all different. And still trying to – and show them – bring it back, like, go out and collect it with the younger ones and the older ones, bring them back.”	Q25. How much time do you spend… -With someone who has cultural knowledge (Elder or knowledge holder)? -Learning culture, kinship, and respect? -Passing on cultural knowledge? -Receiving Aboriginal/Torres Strait Islander healing methods (such as traditional healers, bush medicine)? Q27. In the Aboriginal/Torres Strait Islander community where I live now… -There are people with cultural knowledge (cultural bosses or Elders) I can go to or yarn with

### Expert input and prioritisation of items

The process described above strengthened the accuracy, relevance, and cultural appropriateness of the questionnaire items. At first, the Mayi Kuwayu team tried to capture all aspects of culture which may be relevant to health and wellbeing outcomes. However, over time we realised that it would not be possible to incorporate everything within a relatively short questionnaire. Thus, the team began to make decisions about what key items needed to be included, and what could be excluded based on a combination of focus group feedback and expert input on what would be less critical for the questionnaire to measure.

This final step involved a panel of six reviewers with expertise in questionnaire design and Aboriginal and Torres Strait Islander community research, who ranked the questionnaire content according to three categories. Each panel member reviewed each item separately and were asked to rank the questions as ‘A’, ‘B’, or ‘C’, according to their perceived importance:

- **A:** High priority; should be included in the questionnaire if space allows
- **B:** Lower priority; important but not critical
- **C:** Should not be included

The feedback from the panel was compiled into a collated review document. The Mayi Kuwayu team then reviewed the collated feedback and made the final decision on keeping, dropping, or modifying each item in the questionnaire. After consideration of the panel feedback, 75 questions were removed and seven were modified for clarity. A total of 41 questionnaire items address one or more of the six cultural domains identified by Salmon and colleagues [2] (see Supplementary Table 1 [Additional File 1] for details). An online version of the questionnaire is available to be completed by Aboriginal and Torres Strait Islander individuals aged 16 years and above [26].

## Discussion

Mayi Kuwayu is the first of its kind to identify key cultural domains and develop corresponding questionnaire items which are relevant across the diversity of Aboriginal and Torres Strait Islander cultures in Australia, with Indigenous data sovereignty and governance protocols in place. The few national cultural items which do exist outside of Mayi Kuwayu are of limited use due to the relative absence of Aboriginal and Torres Strait Islander people involvement in their development.

The National Aboriginal and Torres Strait Islander Social Survey (NATSISS), which was last administered in 2014–2015, has six items which relate to Aboriginal and Torres Strait Islander cultures and languages: identification with an Aboriginal and/or Torres Strait Islander tribal group, language/regional group, clan, or mission; recognition of homelands/traditional Country; access to homelands/traditional Country; involvement in cultural events or ceremonies; participation in cultural activities; and whether participants could speak or were learning an Aboriginal and/or Torres Strait Islander language [27]. The NATSISS has largely remained unaltered since it was first administered in 1994 [28] and has been criticised for its failure to reflect the breadth and depth of Aboriginal and Torres Strait Islander concepts of health and wellbeing [29].

The 2020 Aboriginal and Torres Strait Islander Health Performance Framework, used by the Department of Health to monitor progress in population health outcomes and determinant, as well as health-care system performance, subsumes these items within the theme ‘Connectedness to country, land and history; culture and identity’ as an indication of community functioning ([30]: Sect. 1.13). While acknowledging the importance of culture, these items do not address many important aspects of Aboriginal and Torres Strait Islander cultures, such as learning and passing on cultural beliefs and knowledge. They also do not provide any indication of the quality of cultural connection for participants beyond a general accounting of ‘time spent’ and number of cultural activities engaged in, and thus lack meaningful utility for communities.

There are some more detailed cultural items which have been developed with Aboriginal people outside of government settings, but these have only been used in small geographical areas or are only relevant to specific cultural groups. The Aboriginal Cultural Engagement Survey (ACES), which aimed to assess engagement with culture for Aboriginal peoples living in semi-urban areas, was generated with the aid of Aboriginal consultants throughout its development [31]. Using a strengths-based framework, ACES was designed to be used in a variety of settings and aimed to explore the impact of cultural engagement on health outcomes. The ACES was expanded and refined over time following reviews by 18 Aboriginal people with professional cultural expertise, resulting in a 21-item questionnaire designed to address both traditional and modern aspects of cultural participation (for full details, see [31]).

The Interplay Project developed and validated a holistic wellbeing framework and a questionnaire to measure the wellbeing of Aboriginal peoples living in remote areas [32]. Partners from community, government, and the sciences worked collaboratively in a ‘shared space’ to design, implement, and interpret their findings, and communicate the outcomes of the project ([32]: 70). Culture, empowerment, and community were identified as three key priorities for research, alongside health, work, and education (for further details of this process, see [33]). Items used for the Interplay questionnaire were drawn from several other questionnaires (for the full list, see [32]: 73), and were refined and modified during reviews by Aboriginal community researchers. This 40-item questionnaire was administered in four remote Aboriginal communities with 842 participants, and demonstrated that cultural factors have both direct and indirect impacts on wellbeing.

The Yawuru Wellbeing Framework is the only existing study to have developed cultural items for a questionnaire within an exclusively Aboriginal-led leadership and governance framework [34]. Based on the Yawuru concept of *mabu liyan*, or the good life, the questions used for the Yawuru Wellbeing Survey are highly context-specific, reflecting the knowledge and intergenerational experiences of Yawuru women and men in Broome, WA. Seven domains contributing to *mabu liyan* were identified by the researchers in partnership with community members: strong family; strong community; connection to culture, Country, and identity; self-determination; health; and material wellbeing; and subjective wellbeing ([34]: 46). Focus groups were then used to select existing cultural items or develop new items based on the seven domains, and potential items were discussed, refined, and validated by the Yawuru community ([34]: 38). The 57-item Yawuru Wellbeing Survey (M Yap 2021, personal communication, 10 August) was then completed by 156 Yawuru people in 2015 (for further information and results, see [34]).

What these studies demonstrate is that Aboriginal and Torres Strait Islander cultures and their influence on health and wellbeing outcomes can be measured in a meaningful way. The Mayi Kuwayu Study is unique in Australia for developing a questionnaire which is able to be nationally relevant, meaningful, and useful across the diversity of Aboriginal and Torres Strait Islander cultures and communities.

There were a few limitations to the research processes detailed here. The first is that there was a general under-representation of younger people (aged 16–24 years) across the focus groups, and when they were present, they often deferred to older members of the group as per cultural protocols. While the focus groups were not intended to represent all Aboriginal and Torres Strait Islander lived experiences, given that the estimated median age across the Aboriginal and Torres Strait Islander population is 20.3 years [35], the experiences of young people may not have been adequately captured during the focus groups, and thus the final questionnaire content. Second, the duration of the focus groups and the time it took to complete the questionnaire drafts may have had an impact on the type and depth of feedback given. As discussions about culture and wellbeing typically took around an hour, the participants may have been fatigued as they were filling out the draft questionnaire, and thus less inclined to any raise issues they may have had. If we were to run these sessions in the future, we would seek to organise two different types of focus groups, with one discussing culture and its impact on health and wellbeing in the local community, and the other to field test the questionnaire.

## Conclusion

Culture is foundational to Aboriginal and Torres Strait Islander health and wellbeing, but this has rarely been explored in any depth by national epidemiological surveys in Australia. The Mayi Kuwayu Study is the first in Australia to be developed by, with, and for Aboriginal and Torres Strait Islander people, and seeks to reveal the importance of culture for health and wellbeing outcomes. Developing culturally relevant questionnaire items requires a substantial investment of time and resources by researchers and community members. The time and care taken by our team and participating communities has resulted in a questionnaire which speaks to the diversity of Aboriginal and Torres Strait Islander experiences across Australia and addresses key areas where large-scale data is needed as identified by Aboriginal and Torres Strait Islander communities and organisations.

The focus group method was applied in a culturally responsive way, using the shared Indigeneity of the researchers and participants to discuss important aspects of culture and build on the questionnaire content in an iterative process. The *Cultural knowledge and practice* section was presented as an example of how focus group feedback informed the iterative development of items in the questionnaire. Input from experts in Aboriginal and Torres Strait Islander health enabled the Mayi Kuwayu team to further refine the domains and items to be used for the questionnaire. The six cultural domains identified by an international literature review conducted by Salmon and colleagues [2] were consistently raised across the 28 focus groups organised for the Mayi Kuwayu study, and these discussions were instrumental in the development and refinement of questionnaire items.

This paper has demonstrated that it is possible to use a qualitative method, such as focus groups, to inform the development of a quantitative instrument, such as a questionnaire, in epidemiology. Further, it is possible to apply this process to identify key domains of culture which are fundamental to the health and wellbeing of Aboriginal and Torres Strait Islander peoples. These findings have significant implications for the study of cultural determinants of health alongside social determinants in the field of epidemiology and public health. Based on our inclusion of cultural items, data collected by Mayi Kuwayu will provide a significant contribution to the literature on the connections between health and wellbeing and cultural belonging for Aboriginal and Torres Strait Islander peoples [1, 2, 7, 20, 36–42]. Further, these data are available for Aboriginal and Torres Strait Islander community groups and organisations to access and use to develop specific local-level cultural and research initiatives, and grow community wellbeing and connections. Mayi Kuwayu supports the strength and resilience of Aboriginal and Torres Strait Islander communities and will continue to work towards the improvement of our collective health and wellbeing by making our cultures count.

## Supplementary Information

**Additional file 1: Supplementary Table 1. **List of items in the Mayi Kuwayu questionnaire relating to each cultural domain.

## Data Availability

Researchers and organisations seeking to support positive change in Aboriginal and Torres Strait Islander health and wellbeing are encouraged to apply for use of Mayi Kuwayu questionnaire items and data. More information about the study and data access is available through our website (www.mkstudy.com.au).

## Abbreviations

ABS: Australian Bureau of Statistics
ACCHO: Aboriginal Community Controlled Health Organisation
ACES: Aboriginal Cultural Engagement Survey
ACT: Australian Capital Territory
AIATSIS: Australian Institute of Aboriginal and Torres Strait Islander Studies
AIHW: Australian Institute of Health and Welfare
AUD: Australian Dollar
NATSISS: National Aboriginal and Torres Strait Islander Social Survey
NGO: Non-Government Organisation
NSW: New South Wales
NT: Northern Territory
QLD: Queensland
SA: South Australia
TAS: Tasmania
WA: Western Australia
WHO: World Health Organisation
