# Supplementary material for: Developing Aboriginal and Torres Strait Islander cultural indicators: an overview from Mayi Kuwayu, the National Study of Aboriginal and Torres Strait Islander Wellbeing
Source: Int J Equity Health. 2022 Aug 17;21:109. doi: 10.1186/s12939-022-01710-8 (PMC9386936; doi:10.1186/s12939-022-01710-8)
Supplement: Supplementary file 1 — Additional file 1: Supplementary Table 1. List of items in the Mayi Kuwayu questionnaire relating to each cultural domain. [file 12939_2022_1710_MOESM1_ESM.docx]

**Supplementary Table 1** List of items in the Mayi Kuwayu questionnaire relating to each cultural domain

| **Cultural Domain** | **Questionnaire Item** | **Item Detail** |
| --- | --- | --- |
| Connection to Country | 7*, 8*, 9*, 10*, 11*, 12*, 13, 14*, 92 | 13. What is the name of the suburb, community, outstation, town or Island where you currently live? [free text box]  92. Have you ever participated in: *Select all that apply.*   1. Deadly Runners or Indigenous Marathon Project 2. Koori or Murri Knockout 3. Traditional Owner Group 4. Ranger program 5. Indigenous Protected Areas 6. Native Title Group 7. Family Wellbeing Program 8. Deadly Choices 9. Language program 10. NDIS 11. Any Quit Smoking program, service, or activity 12. ANFPP 13. None of these |
| Beliefs and knowledge | 12*, 15*, 16*, 25*, 92, 103* | 92. As above. |
| Language | 19*, 20*, 21*, 22*, 23*, 24*, 92 | 92. As above. |
| Family, kinship, and community | 4*, 5*, 11*, 18*, 22*, 23*, 25*, 33, 34, 35, 43, 56, 57, 75, 84, 85, 89, 92, 93*, 94*, 95, 101, 102, 103*, 104*, 105, 106 | 33. How many people live with you?  34. How many children live with you?  35. Who lives with you? *Select all that apply.*   1. No one 2. My partner or spouse 3. My child(ren) 4. My grandchild(ren) 5. Someone else’s children 6. My parent(s) 7. My grandparent(s) 8. My sibling(s) 9. My cousin(s) 10. My Aunty(ies) or Uncle(s) 11. Other family (in-laws) 12. Other friends or visitors   43. Which words best describe your family's money situation?   1. We have lots of savings 2. We have some savings 3. We have just enough money to get us to the next payday 4. We run out of money before payday 5. We are spending more than we get 6. Unsure   56. Where do you usually go for health care (non-urgent)? *Select all that apply.*   1. Aboriginal Medical Service or Community Controlled Health Service 2. GP clinic 3. Hospital 4. Traditional Healer 5. Unsure 6. Other: [free text box]   57. If you could choose, where would you go for health care (non-urgent)? *Select all that apply.* [responses as 56 above]  75. Why do you want to quit [smoking]? *Select all that apply.*   1. Advertising against smoking 2. Medical advice 3. My health 4. Health of my family 5. Cost 6. Pregnancy 7. Too many non-smoking areas 8. Pressure from family or friends 9. Other: [free text box]   84. What led you to quit? *Select all that apply.* [responses as 75 above]  85. What helped you quit? *Select all that apply.*   1. Smoking program 2. Quitline 3. Online support 4. Health professional 5. Family or friends 6. Patches, gum, inhaler (NRT) 7. Stop smoking medication 8. Quit on my own 9. Other: [free text box]   89. How much do you agree? [response options: Not at all, A little bit, A fair bit, A lot]   1. Non-smokers miss out on gossip or yarning. 2. My community disapproves of smoking. 3. Smoking is not that risky.   92. As above.  95. How often do these things happen to you? [response options: Not at all, A little bit, A fair bit, A lot]   1. I am treated with less respect than other people. 2. I receive worse service than other people (including at restaurants, stores, Centrelink, housing). 3. People act like I am not smart. 4. People act like they are afraid of me. 5. I am called names, insulted, or yelled at. 6. I am followed around in shops. 7. I am watched more closely than others at work or school. 8. Police unfairly bother me.   101. In the last year, has any one in your close family… [response options: No, Yes, Not relevant]   1. Been badly hurt or sick? 2. Passed away? 3. Lost a job? 4. Had problems at work? 5. Had an alcohol or drug problem? 6. Experienced violence? 7. Had stuff stolen? 8. Been arrested or been in youth detention or prison? 9. Had a relationship break up (split up)? 10. Had children taken away?   102. Are any of these a problem where you live? [response options: Not at all, A little bit, A fair bit, A lot, Unsure]   1. Drinking too much grog? 2. Tobacco smoking? 3. Drugs? 4. Sniffing? 5. Racism? 6. Gambling? 7. Family violence? 8. People fighting or not getting along? 9. Humbugging (people wanting stuff all the time)?   105. Do you often care for a sick or disabled family member or friend? [response options: No, Yes]  106. If yes, how many hours each week do you usually spend caring for this person? [response options: XX hours per week, Unsure] |
| Cultural expression and continuity | 3*, 12*, 15*, 16*, 17*, 25*, 26*, 92, 103* | 92. As above. |
| Self-determination and leadership | 27*, 48, 49*, 92, 93*, 94* | 48. How satisfied are you with your life? [response options: Not at all, A little bit, A fair bit, A lot]  92. As above. |

* A licence is required from the Mayi Kuwayu Data Governance Committee to use these items, and so they are not detailed here.
